# Central Precocious Puberty (CPP) in Two Girls With Autism Spectrum Disorder (ASD)

**DOI:** 10.7759/cureus.35671

**Published:** 2023-03-01

**Authors:** Yuko Moriuchi, Tatsuo Fuchigami, Mio Horie, Ryutaro Yamada, Ichiro Morioka

**Affiliations:** 1 Pediatrics, IMS Fujimi General Hospital, Saitama, JPN; 2 Pediatrics and Child Health, Nihon University School of Medicine, Tokyo, JPN

**Keywords:** secondary sexual characteristics, psychosocial burden, gnrh analog therapy, central precocious puberty, autism spectrum disorder (asd)

## Abstract

In recent years, some cases of central precocious puberty (CPP) have been reported in patients with autism spectrum disorder (ASD). Here, we report CPP in two girls with ASD. The first case was a girl, 7 years and 9 months of age. Breast budding was observed at 7 years and 2 months and pubic hair at 7 years and 8 months of age. She was diagnosed with CPP based on guidelines and ASD according to her developmental history. Considering the psychosocial burden caused by the discrepancy between her cognitive and behavioral development and the progression of secondary sex characteristics, gonadotropin-releasing hormone (GnRH) analog therapy was initiated. Case 2 was a girl, 9 years and 8 months of age. She was diagnosed with ASD according to her developmental history. Treatment using oral aripiprazole for hypersensitivity to touch and taste had been initiated, with the onset of menarche at 9 years and 10 months of age. Breast budding had been observed before 7 years and 6 months old. She was diagnosed with CPP based on guidelines. Considering that menarche was not a significant psychosocial burden and the difficulty for her and her family to attend regular follow-ups, GnRH analog therapy was not initiated. Although the pathophysiological pathway linking ASD and CPP remains to be elucidated clinically, attention to CPP in ASD is needed, considering the increase in reported cases. In addition, the indication of GnRH analog therapy should be judged considering the psychosocial burden associated with secondary sexual characteristics.

## Introduction

The contribution of genetic factors to precocious puberty has been previously reported [1-3]. Of these, an association between autism spectrum disorder (ASD) and central precocious puberty (CPP) has been suggested [4-7]. Herein, we describe two cases of CPP associated with ASD.

## Case presentation

**Figure 1:**
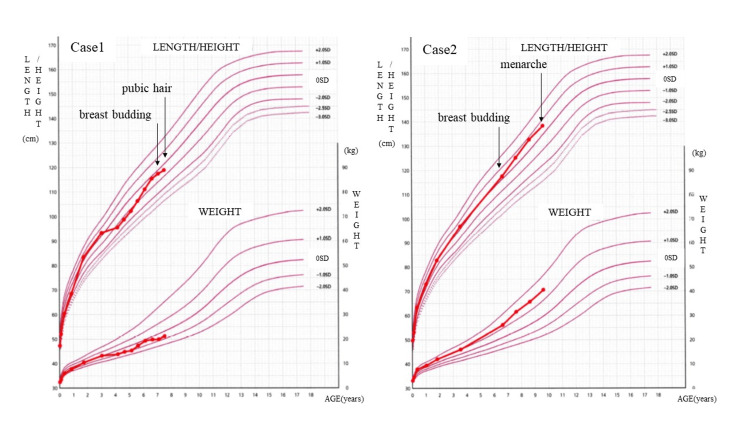
Growth charts for Cases 1 and 2. For Case 1, breast buds at 7 years and 2 months and pubic hair at 7 years and 8 months of age. For Case 2, breast budding before 7 years and 6 months and menarche at 9 years and 10 months of age.

The first case was a girl, 7 years and 9 months of age, who presented to our hospital for assessment. Her breast budding had been observed at 7 years and 2 months and pubic hair at 7 years and 8 months of age (Figure 1, Case 1). Her medical history was unremarkable. Her developmental history revealed poor communication skills, auditory hypersensitivity, and anxiety in approaching novel situations. Her diet was reported to be imbalanced. There was no family history of precocious puberty. Her target height, calculated by her parent’s height, was 159 cm. On admission, anthropometric measurements were as follows: height, 128.7 cm (+1.1 standard deviation, SD), and weight, 30.9 kg (+1.4 SD). The Tanner pubertal stage was II-III for breast development and II for pubic hair. Hormone serum levels were as follows: estradiol (E2), 14 pg/mL; luteinizing hormone (LH), 0.40-12.0 mIU/mL; follicle-stimulating hormone (FSH), 9.13-29.27 mIU/mL after gonadotropin-releasing hormone (GnRH) loading, which was pubertal levels (Table 1). Based on the bone age guidelines using the radius, ulna, and short finger bones (RUS), her bone age was evaluated at 10 years, which was more advanced than her chronological age. No abnormalities in brain structure were noted on MRI without contrast. Based on the guidelines for CPP, two main symptoms (breast enlargement before 7 years and 6 months and pubic hair development under 8 years old), promotion of bone maturation, excessive pituitary gonadotropin secretion, and increased secretion of sex steroid hormones were observed, and other diseases were excluded; thus, CPP was diagnosed. She was also diagnosed with ASD due to impaired social communication, hyperacusis, and fixation on identity. It was medically necessary and considering the psychosocial burden caused by the discrepancy between her cognitive and behavioral development and the progression of secondary sex characteristics, GnRH analog therapy was initiated. After explaining to the parents that treatment has the advantages of delaying menarche, subcutaneous injection of leuprorelin acetate every 4 weeks was started at 7 years and 11 months of age. She is now 8 years and 6 months old but has not yet had menarche and gained height.

**Table 1:** Baseline and hormonal parameters. E2, estradiol; PRL, prolactin; LH, luteinizing hormone; FSH, follicle-stimulating hormone; GnRH, gonadotropin-releasing hormone

Parameter	Case 1	Case 2	Reference range
Serum E2	14 pg/mL	45 pg/mL	<12 pg/mL
Serum PRL	Not examined	13.56 ng/mL	<15 ng/mL
Serum LH (basal)	0.40 mIU/mL	1.3 mIU/mL	0.01-0.09 mIU/mL
Serum LH (after GnRH)	12.0 mIU/mL	Not examined	1.93-4.73 mIU/mL
Serum FSH (basal)	9.13 mIU/mL	1.91 mIU/mL	0.54-2.47 mIU/mL
Serum FSH (after GnRH)	29.27 mIU/mL	Not examined	0.97-6.31 mIU/mL

Case 2 was a girl, 9 years and 8 months of age, who presented to our hospital for assessment. Her diet was reported to be imbalanced. She was diagnosed with ASD due to revealed poor communication skills, pervasive attention to detail, proneness tantrums, and hypersensitivity to touch and taste. Treatment using oral aripiprazole (dose, 0.5-1.0 mg/day) for hypersensitivity to handling and taste was initiated at 9 years and 8 months of age. After the start of aripiprazole therapy, the patient tolerated clothing and went outdoors, with the onset of menarche at the age of 9 years and 10 months. According to the mother, breast budding had been observed before 7 years and 6 months of age; thus, precocious puberty was suspected (Figure 1, Case 2). There was no family history of precocious puberty. Her target height, calculated by her parent’s height, was 157 cm. On admission, anthropometric measurements were as follows: height, 140 cm (+0.65 SD), and weight, 40.0 kg (+1.2 SD). The Tanner stage was II-III for breast development and II for pubic hair. Hormone serum levels were as follows: E2, 45 pg/mL; prolactin (PRL), 13.56 ng/mL; basal LH, 1.3 mIU/mL, FSH, 1.91 mIU/mL; and LH/FSH ratio, 0.68 (Table 1). Her bone maturity age was estimated at 12 years, using the RUS method, which was more advanced than chronological age. She could not get MRI because of her auditory hypersensitivity, but no abnormalities in brain structure were noted on CT imaging. Based on the guidelines for CPP, two main symptoms (breast enlargement before 7 years and 6 months and menarche before 10 years and 6 months of age), promotion of bone maturation, excessive pituitary gonadotropin secretion, and increased secretion of sex steroid hormones were observed, and other diseases were excluded; therefore, CPP was diagnosed. As it was expected to have poor compliance with the medication and follow-ups, GnRH analog was not initiated with her mother’s consent.

## Discussion

In recent years, some reports of CPP have been reported in children with ASD [4-7]. David and Mark [7] reported that the precocious puberty rate was 3.15 times higher in the ASD group than in the control group and that precocious puberty was more likely to occur after three years of age. An interaction between acquired factors, such as gene susceptibility and environmental factors, might be an underlying mechanism.

Regarding acquired factors, significantly higher levels of salivary androgens have been reported in older children with ASD than in age-matched controls, indicative of precocious adrenarche [8-9]. However, to date, a pathway connecting ASD to CPP has not been clarified.

Another factor that causes menstrual abnormalities in ASD is medication. However, these second-generation antipsychotics have a high risk for metabolic and endocrine side effects, such as hyperprolactinemia [10]. Hyperprolactinemia is reported to have a risk of amenorrhea, enlarged mammary glands, and future osteoporosis, especially in girls [11-12]. In addition, administration of aripiprazole when PRL concentration is high, such as in combination with other antipsychotics, may decrease PRL concentration and cause menorrhagia [11, 13]. In our case 2, since menstruation occurred during aripiprazole administration, the PRL value was measured considering abnormalities in PRL concentration due to drug properties, but it was normal. According to a report by Okumura et al. [14], only a few percent of children and adolescents whose PRL values were measured during the first year of antipsychotic treatment. However, since there is a risk of menstrual irregularities due to antipsychotic administration, evaluation including PRL is necessary. As a limitation of this report, we only described two cases of CPP with ASD; therefore, accumulating more cases is necessary.

## Conclusions

Here, we reported CPP in two girls with ASD. Although the pathophysiological pathway linking ASD and CPP remains to be elucidated clinically, attention to CPP in ASD is necessary, considering the increase in reported cases.
